# Bibliometric analysis of research on gene expression in spinal cord injury

**DOI:** 10.3389/fnmol.2022.1023692

**Published:** 2022-10-31

**Authors:** Siqiao Wang, Weijin Qian, Shaofeng Chen, Shuyuan Xian, Minghao Jin, Yifan Liu, Hao Zhang, Hengwei Qin, Xinkun Zhang, Jiwen Zhu, Xi Yue, Chaofeng Shi, Penghui Yan, Runzhi Huang, Zongqiang Huang

**Affiliations:** ^1^Department of Orthopedics, The First Affiliated Hospital of Zhengzhou University, Zhengzhou, China; ^2^Division of Spine, Department of Orthopedics, Tongji Hospital Affiliated to Tongji University School of Medicine, Shanghai, China; ^3^Tongji University School of Medicine, Shanghai, China; ^4^Shanghai Jiao Tong University School of Medicine, Shanghai, China; ^5^Department of Orthopaedic Surgery, The First Affiliated Hospital of Naval Medical University, Shanghai, China; ^6^Department of Orthopedics, Naval Medical Center of PLA, Second Military Medical University Shanghai, Shanghai, China; ^7^Department of Burn Surgery, The First Affiliated Hospital of Naval Medical University, Shanghai, China; ^8^Research Unit of Key Techniques for Treatment of Burns and Combined Burns and Trauma Injury, Chinese Academy of Medical Sciences, Shanghai, China

**Keywords:** spinal cord injury, gene expression, mechanism, microarray, sequencing

## Abstract

**Background:**

Spinal cord injury (SCI) is a severe disease with motor and sensory function being destroyed, which leads to a poor prognosis and a serious financial burden. It is urgent to figure out the molecular and pathological mechanisms of SCI to develop feasible therapeutic strategies. This article aims to review documents focused on gene expression in SCI and summarize research hotspots and the development process in this field.

**Methods:**

Publications of SCI-related studies from 2000 to 2022 were retrieved from the Web of Science Core Collection database. Biblioshiny was used to evaluate the research performance, core authors, journals and contributed countries, together with trend topics, hotspots in the field, and keyword co-occurrence analysis. Visualized images were obtained to help comprehension.

**Results:**

Among 351 documents, it was found that the number of annual publications increased in general. The most productive country was China, followed by the United States with the highest influence and the most international cooperation. *Plos One* was the journal of the maximum publications, while *Journal of Neuroscience* was the most influential one. According to keyword co-occurrence and trend topics analysis, these articles mainly focused on molecular and pathological mechanisms as well as novel therapies for SCI. Neuropathic pain, axonal regeneration and messenger RNA are significant and promising research areas.

**Conclusion:**

As the first bibliometric study focused on gene expression in SCI, we demonstrated the evolution of the field and provided future research directions like mechanisms and treatments of SCI with great innovativeness and clinical value. Further studies are recommended to develop more viable therapeutic methods for SCI.

## Introduction

The spinal cord is responsible for connecting the brain with peripheral nerves entering or exiting the cord, and it can produce reflexes which also receive the regulation of the brain at the same time (Silva et al., 2014). When the spinal cord is torn or compressed, or the blood vessels supplying the spinal cord infarct suddenly due to traffic accidents or other disasters, it will lead to spinal cord injury (SCI), which is a severe disease and cannot be cured even nowadays (DeVivo et al., 2002; Smith et al., 2005). SCI results in temporary or permanent disability in a variety of organs throughout the body with motor and sensory function being destroyed, which not only has bad impacts on the patient’s mental and social well-being state but also exerts a huge financial burden on their family (Yuan et al., 2018; Shi et al., 2019). The incidence of SCI has been retained by 26.5 cases per million people over the past decade (Barbiellini Amidei et al., 2022). Nowadays, the disease still lacks effective treatment options which can only use palliative treatments, such as preventing injury progression, deafferentation pain syndromes, and managing complications (Singh et al., 2010; Garcia-Rill et al., 2012). Thus, it is urgent to understand the pathological and molecular mechanisms of the SCI in order to develop feasible therapeutic strategies that can overcome difficulties in clinical treatments and rehabilitation.

Gene expression characteristics of SCI are a burgeoning research direction, and cells in different anatomical parts possess unique gene expression profiles. It can be studied using transcriptomics or proteomics or a combination of the two omics (Haider and Pal, 2013). A transcriptome represents all types of RNA presented in a cell, and in transcriptome analysis, microarray analysis and RNA-sequencing (RNA-seq) are two major techniques. Importantly, with the techniques introduced above or others, some scholars have reported that up or down regulation of different gene expression leads to activation or suppression of specific molecular and pathological mechanisms and pathways after SCI, which was also related to patient prognosis. Extracellular signal-regulated kinases (ERKs), for instance, interacted with growth factors by phosphorylating transcription factor ELK-1, which was involved in regulating growth of axon (Wen et al., 2016). Another study reported that suppressing the triggering receptor expressed on myeloid cell 1 (TREM1), which was highly expressed in SCI tissues of mice, helped reduce inflammation and oxidative stress regulated by heme-oxygenase-1 (HO-1), and significantly improved the SCI outcomes (Li et al., 2019). These mechanisms will become research hotspots in the future, providing new ideas for potential trauma treatment. Therefore, it is necessary to conduct a bibliometric analysis of the field of gene expression in SCI.

Notably, there is an increasing number of articles being published every year on gene expression in spinal cord injury. Hence, it is imperative for researchers to keep an appreciable and constant update of the latest researches. Bibliometric analysis is a kind of quantitative analytic approach that depicts the knowledge structure and development trends in a specific research field with science mapping, which is widely used to review and summarize the existing researches in a given field (Mou et al., 2019). With the application of mathematical statistics, it enables statistical analysis of humongous volumes of information to acquire an overall understanding of a certain field, such as the identification of influential authors, countries, journals and affiliations, illustrating hotspots and trending topics over time. Bibliometric analysis has gained huge popularity in multidisciplinary fields recently. For example, a novel bibliometric study on Apache Hadoop investigated the statistical characteristics of collected publications, and it also summarized the research hotspots as well as future trends in this field through keyword analysis (Zhang and Lin, 2022). Bibliometric methods were also applied in the field of wastewater treatment and emerging contaminants, which discussed the intellectual structure, collaboration network, and development directions of this field, while the removal technology and high-efficiency detection were hotspots at present (Chen et al., 2022). Its popularity can be attributed to the availability, advancement, and accessibility of bibliometric software such as Citespace, VOSviewer, R-bibliometrix, and scientific databases such as Web of Science (WOS), and the cross-disciplinary pollination of the bibliometric methodology including deep learning methods for image processing (He et al., 2022). Importantly, the popularity of bibliometric analysis is not a fad but rather a reflection of its use for dealing with large volumes of scientific data, and providing international academic collaborations and trend topics in a given field. So far, bibliometrics has been used in research on gene expression features in various diseases, which all showed great impact. It was shown that mesenchymal stem cell-derived microRNA was the frontier in myocardial infarction research (Zhou et al., 2018). Another bibliometric analysis with enormous practical significance focused on the gene expression profiles of COVID-19 and summarized highly expressed genes like IL-6, TNF, ACE2, and TMPRSS2, which offered solid evidence for researchers to explore novel therapeutic targets (Wu et al., 2021). However, the application of bibliometrics in the field of gene expression in SCI still remains blank. Herein, we performed a bibliometric analysis to provide an overview of the knowledge structure and research trends concerning the gene expression of SCI in the past 20 years.

Therefore, with innovation and clinical value, we evaluated multiple aspects like authors, countries, and journals and also discussed the evolution and hot topics of this field based on bibliometric methods, in order to help scholars establish a framework of the whole field and obtain an overview of the developing process in recent years. The study may also predict the research hotspots in the future. Besides, it can help the medical system carry out more effective and high-quality research on therapeutic measures for patients at the same time.

## Materials and methods

### Data sources and retrieval strategies

The Web of Science (WOS) is the greatest global database for the collection and retrieval of multiple publications from various academic disciplines. Searches were conducted based on the WOS Core Collection database to obtain literature in the Science Citation Index Expanded (SCI-EXPANDED) on 27 April 2022. The retrieval strategies were as follows: {[(TS = spinal cord injury) OR (TS = spinal cord injuries)) AND ((TI = transcriptomic) OR (TI = proteome) OR (TI = proteomics) OR (TI = metabolomics) OR (TI = bioinformatics) OR (TI = metagenomics) OR (TI = metatranscriptomics) OR (TI = omics) OR (TI = microarray) OR (TI = sequence) OR (TI = RNA-seq) OR (TI = sequencing) OR (TI = ATAC-seq) OR (TI = single cell sequencing) OR (TI = single cell sequence) OR (TI = single cell RNA sequencing) OR (TI = single cell RNA sequence) OR (TI = expression profile) OR (TI = bioinformatic*) OR (TI = high throughput)]} were the search term, while mainly articles and reviews in English, primarily from 1 January 2000 to the day of retrieval, may be included. Some publications were excluded after screening by title and abstract. Finally, a total of 351 studies were included, and all data were exported with full records and references in plain text format, and were then imported into the bibliometrics analysis software for further analysis. (See Supplementary Data Sheet 2 in Supplementary material for details).

### Methodology

With the development of technology, the results of graphics and visualization can be utilized to illustrate data and display the interrelationship of different types of information, making findings more comprehensive (Huang et al., 2022). VOSviewer and Citespace are tools mainly used in large amounts of knowledge mapping and visualization analysis of scientific literature (Chen, 2006; Van Eck and Waltman, 2010; Pan et al., 2018). Bibliometrix is a unique open-source tool for performing bibliometric analysis, comprehensive visualization, and knowledge mapping analysis (Aria and Cuccurullo, 2017a). And biblioshiny, which provides a web-based operator interface for bibliometrics, was used to analyze the data collected from the WOS. It could provide a quantitative assessment of enormous volumes of scientific data, such as publication and citation data, to evaluate research performance, maturity, growth, core authors and journals, and trends of a research community (Aria and Cuccurullo, 2017b). In addition, biblioshiny could build matrices for co-citation, collaboration, co-word analysis and coupling, and could provide clear images and graphics for visualization (Derviş, 2019). The detailed code of biblioshiny was shown in Supplementary Data Sheet 3 in Supplementary material.

As for the bibliometric methods, citation analysis is one of the most indispensable approaches in bibliometric analysis. A citation means the acknowledgment that one article receives from another, and it also represents that there is a strong relationship between the cited and citing items, while the strength of this relationship can be measured by the number of citations to each other (Osareh, 1996). The citation analysis is a probable unbiased evaluation of the correlation of documents, authors, journals, countries, and so on, and it can be used for information retrieval and summarization of the topical structure of various fields. Bibliographic coupling analysis and co-citation network analysis are two different methods for analyzing closely related documents based on citation analysis. The bibliographic coupling refers to two papers which have cited the same reference, and this reference is defined as a unit of the coupling between the two papers (Kessler, 1963). Co-citation means the frequency with which two papers are cited at the same time, while a co-citation network can be achieved in a particular scientific field (Small, 1973). Both clustering analytic methods described above are useful tools to monitor the development of a certain specialty, identify the popular research topics, and assess the interrelationships between documents. Collaboration network analysis focuses on the cooperation and connection of authors, countries, affiliations, and journals, which builds a structured collaborative map in a particular field (Cross et al., 2002). Co-word analysis is also employed in this article to identify the research content and the development of the field over time (Callon et al., 1991). It measures the association strength and correlation of co-occurred keywords within the same document, indicating a relation between the topics to which they refer (Cambrosio et al., 1993). It gives a visualization of the conceptual structure in a specific discipline (Ding et al., 2001).

H-indices are used to evaluate a scholar’s scientific influence and outputs in a concise and useful manner. It means that for each researcher, h of his documents have at least h citations, while his other documents have less than h (Hirsch, 2005). This computable index provides an estimation of the broad scientific impact of a researcher in an evenhanded way.

## Results

### Annual publication

**Figure 1:**
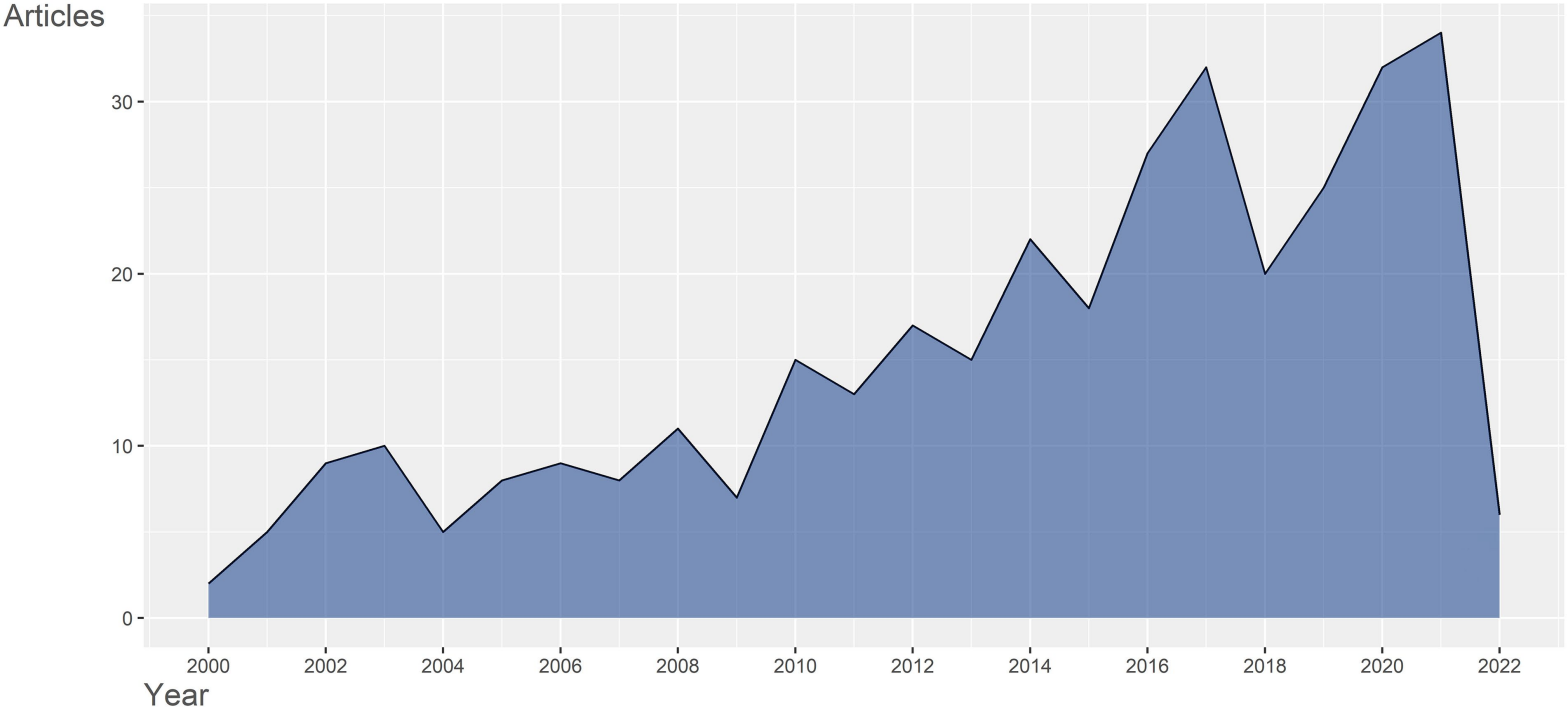
Annual publications of gene expression in spinal cord injury from 2000 to 2022.

351 documents retrieved from 2000 to 2022 were written by 2,127 authors from 42 countries and regions, published in 199 journals, and cited 16,594 references. The number of publications published each year can reflect the popularity of research in a particular field. As was shown in Figure 1, the annual publication outputs have increased from 2000 to 2021 generally. Before 2010, the annual publication output was less than 10, except 2008 in the field of gene expression in SCI. From 2010 to 2021, overall, with more research being undertaken in the field, the number of publication outputs grew explosively and reached 34 in 2021. There were six articles issued during the first quarter of 2022. A substantial association between publication output and publication year was identified by utilizing polynomial model fitting (Coef R: 0.863 for articles). The publication output for articles is predicted to reach around 30 in 2022, based on the polynomial curve fitting (Supplementary Figure S1A). The number is relatively the same as that in the previous year, indicating that this field is still gaining attention continuously. The growing trend demonstrated that gene expression in SCI was gaining traction and had huge development potential in both basic and clinical experiments. The peaks occurred in 2017 and 2021 implicated important research outcomes acquired, respectively. For instance, in 2017, the pathogenesis of neuropathic pain associated with SCI was a hot topic. A study investigated the non-coding RNAs (ncRNAs) expression profiles in the spinal cord after spared nerve injury (SNI) induced neuropathic pain through a sequencing approach, and the downstream pathways connected with those ncRNAs in neuropathic pain pathogenesis, such as ribosome, the PI3K-Akt signaling pathway and focal adhesion, were predicted. It also constructed a regulatory network of ncRNA, miRNA, and mRNA for the first time (Zhou et al., 2017). A bioinformatic study related to chronic neuropathic pain following SCI identified 592 differentially expressed genes and 209 significantly activated pathways in the process of neuropathic pain, among which Ccl3 and MAPK were the most up-regulated gene and the most activated pathway (Zhang and Yang, 2017). The increased Ccl3 activated its cytokine receptor, CCR5, leading to p38 MAPK activation in microglia in the spinal cord, which induced the release of inflammatory mediators, aggravating the neuropathic pain (Ji and Suter, 2007). Therefore, inhibiting the expression of Ccl3 and p38 MAPK signaling pathway after SCI might be a novel therapeutic strategy for neuropathic pain. Another study detected a crucial protein, Ctnnb1, with the highest connectivity degree by protein–protein interaction network in injured dorsal root ganglia (DRG; Chen et al., 2017). Ctnnb1 participated in the Wnt/β-catenin pathway in promoting neural development, regeneration of axons and functional recovery of neuropathic pain after SCI, which also contributed to the research on molecular targets of SCI (Suh et al., 2011; Gao et al., 2016). In 2021, a study identified three clusters of neurons in the dorsal root ganglia in SNI-induced neuropathic pain by single cell RNA sequencing technology and demonstrated the dynamic neuron type changes in the development of neuropathic pain. This transcriptomic analysis also depicted that cardiotrophin-like cytokine factor 1 increased in the second neuron cluster, which promoted the SNI-induced mechanical allodynia, revealing the molecular mechanisms of neuropathic pain (Wang et al., 2021). A recent study that applied proteomic analysis observed that the upregulated proteins in acute and subacute phases of SCI were correlated with the complement and coagulation cascades, immune response, cholesterol metabolism, and lysosome pathway (Yao et al., 2021). For example, as one of cathepsins, the expression of CTSB increased after SCI, acting as a driver of neuroinflammation through lysosome pathway (Nakanishi, 2020). In general, the current studies have focused on the gene expression patterns and molecular mechanisms of SCI or other SCI-derived diseases in order to disclose potential targets for subsequent treatments and have already made preliminary achievements.

### Contributions of authors, countries, and affiliations

**Figure 2:**
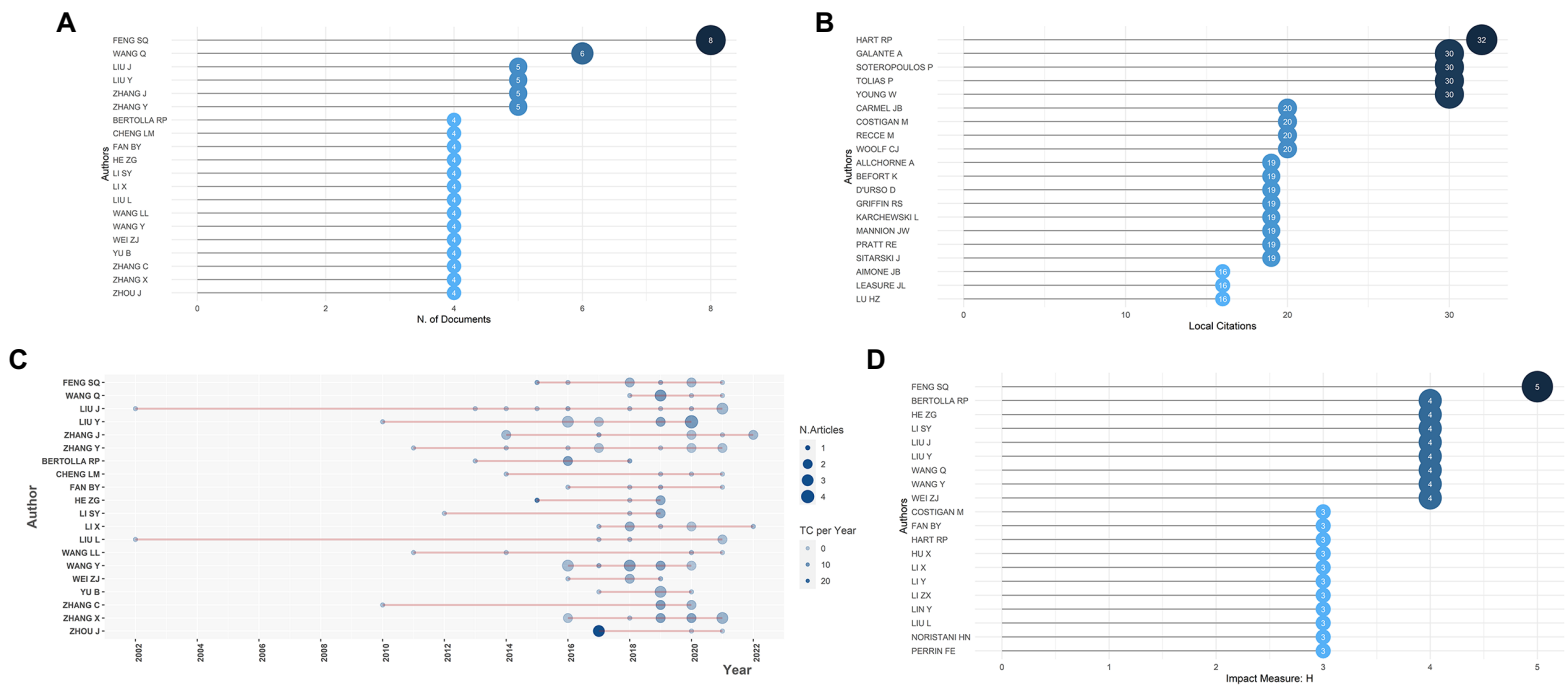
**(A)** The top 20 most relevant authors. **(B)** The top 20 most frequently cited authors. **(C)** The top authors’ production over time; the length of the line represented the author’s research timeline, the size of nodes reflected the publication outputs, and the color density was proportional to total citations per year. **(D)** The authors’ impact as measured by the h-index.

From 2000 to 2022, 2,127 authors have published their research related to gene expression in SCI. Exploring productive and influential authors might help researchers understand active research groups and build direct collaboration with potential research teams. Figure 2A showed the top 20 relevant authors, ranked by the number of publications who met the minimal publication standard of 4, and 19 of them were from China. As presented, the most prolific author, Shi-Qing Feng from Tianjin Medical University General Hospital of China, ranked first with eight articles. Following that, Qian Wang of China’s Huazhong University of Science and Technology contributed six articles, placing second. The rest of the authors published five or four articles, ranging from 3 to 20. The most locally cited author was Hart RP with 32 local citations, and the other four authors’ documents were locally cited more than 30 times in the collection, shown in Figure 2B. According to Lotka’s law shown in Supplementary Figure S1B, only 0.9% of 2,127 authors had published at least four documents. The top authors’ production over time was shown in Figure 2C, in which the length of the line represented the author’s research timeline, the node size reflected the number of publications, and the color density was proportional to total citations per year, demonstrating the long-term contributions of prolific authors to the field of gene expression in SCI. Furthermore, the collaboration network of authors was displayed in Supplementary Figure S1C, which indicated that prolific authors usually cooperated with each other to advance research. With the application of Citespace, the most scientific authors in this field were also recognized (Supplementary Figure S1D), such as Michael Costigan, James W. Fawcett, Damian Szklarczyk, and Paul Shannon. While the latter two introduced several software and tools to construct interaction networks of biomolecules, which was beneficial to studies in the field of gene expression in SCI (Shannon et al., 2003; Szklarczyk et al., 2019), the anterior two contributed directly to this field (Fawcett and Asher, 1999; Costigan et al., 2009).

In order to analyze the scientific influence of authors in the field, they were ranked by h-index (Hirsch, 2005; Figure 2D). The value of h implies that a certain author has produced h articles, each of which has been referenced at least h times (Zhu et al., 2021). Shi-Qing Feng is the most prolific author. His h-index is 5, ranking first among 20 authors. Additionally, citation statistics are taken into account, and there are some fluctuations in the rankings (Figure 2C). For example, Zhigang He had a total of 159 citations from 2015 to 2022 and 19.88 citations per year but ranked 10th based on publication, while Shi-Qing Feng took second place with 106 citations from 2015 to 2022 and 13.25 citations per year. This indicates that authors who have a higher number of citations per year usually produce more highly-cited papers. As illustrated in one of Zhigang He’s documents with 127 cited times, up-regulation of c-myc would promote axon regeneration in axotomized retinal ganglion cells (RGCs), suggesting that c-myc and its downstream pathways can be targeted for neural repair, which might also be applied in neuron regeneration after SCI (Belin et al., 2015). Shi-Qing Feng’s representative work in 2019 depicted the protein profile of Schwann cell-derived exosomes (SCDEs) related to neural restoration, providing ideas for novel treatment strategies (Wei et al., 2019). The dynamic variation of expression of key genes, regulatory factors, proteins and pathways has gradually become a research hotspot in the SCI field, offering orientation for studies of potential therapy for SCI patients.

**Figure 3:**
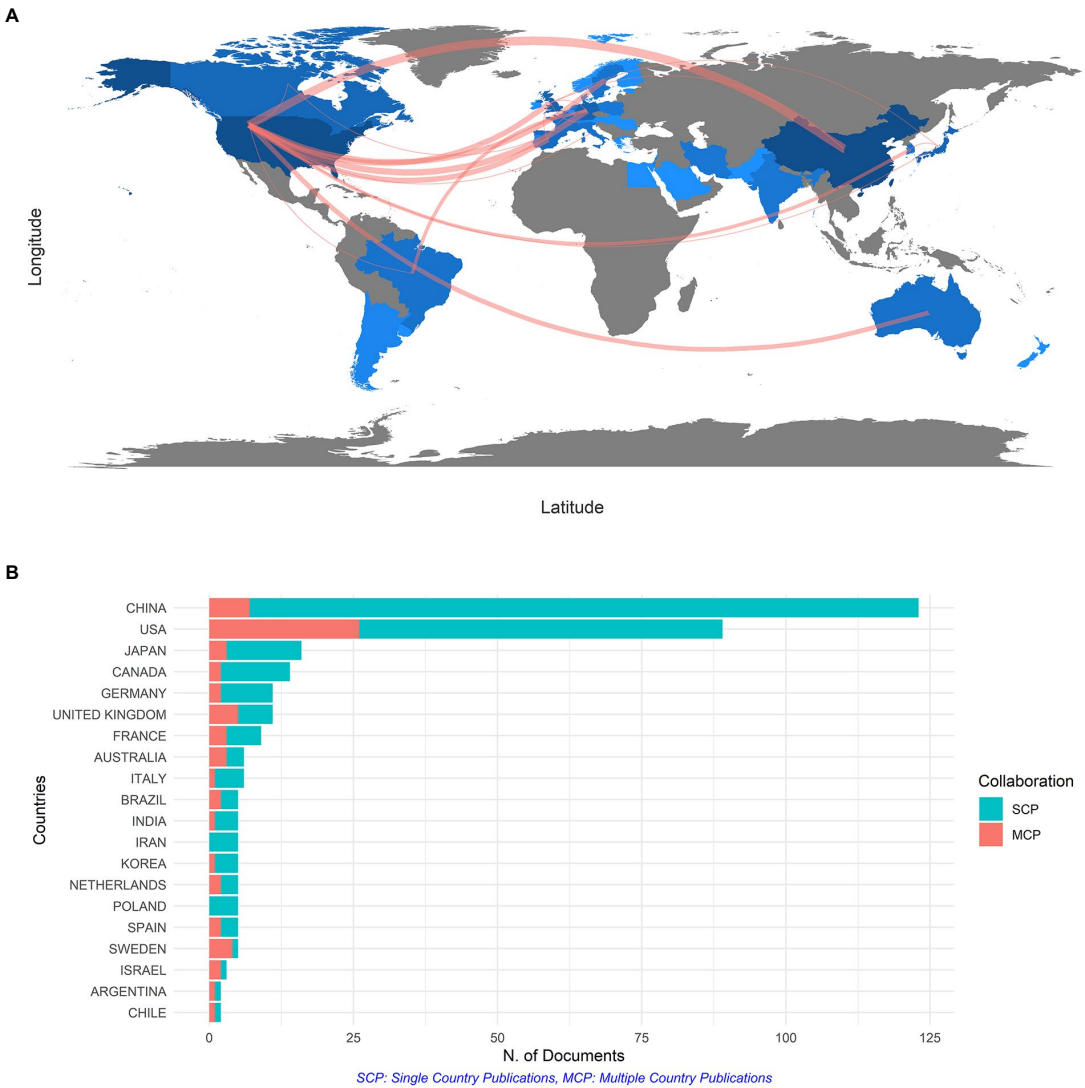
**(A)** The network map of collaboration among countries. **(B)** The top 20 productive countries in terms of the number of documents counted by the corresponding author’s country.

For the sake of identifying the most productive and influential countries, an overview of the number of publications of all 42 countries and regions was presented in Figure 3A, and these countries were spread across five continents. The color shades indicated the number of publications. The darker color of a certain country on the map suggested more participation from it in this field. Supplementary Table S1 listed the top eight countries in terms of the number of publications. China topped the ranking of 389 records cited 1,682 times, followed by the United States with 333 records cited 3,529 times. The top 20 citated countries and regions are shown in Supplementary Figure S2A. In terms of average article citations, the United States ranked first with 39.65 times, followed by the United Kingdom with 37.18 times. However, China only got 13.67 citations. As displayed in Figure 3A, there was a strong link between the United States and China, which were also the top two most productive countries, suggesting that the huge partnership community between the two big countries for gene expression in SCI research has taken shape. Besides, the United States had more connections with other major publishing regions, such as Europe, in the collaborative network map. In Supplementary Figure S2B, China and the United States were identified as the top two most impactful countries by Citespace, with high outputs and strong links between them and other countries. It served as a reference for our analysis of countries and regions. As plenty of authors from China and the United States focused on this field, it reflected that gene expression in SCI was important and promising.

Figure 3B showed the nationality of co-author, the number of single country publications and multiple country publications which indicated the number of co-authored papers with authors of the same nationality or from different countries distinctly. The MCP ratio showed the rate of international collaboration. As predicted, the MCP ratio of the United States was 0.2921, much higher than other countries like China (MCP ratio = 0.0569) and Japan (MCP ratio = 0.1875), which also reflected the high level of cooperation between the United States and other regions around the world.

**Figure 4:**
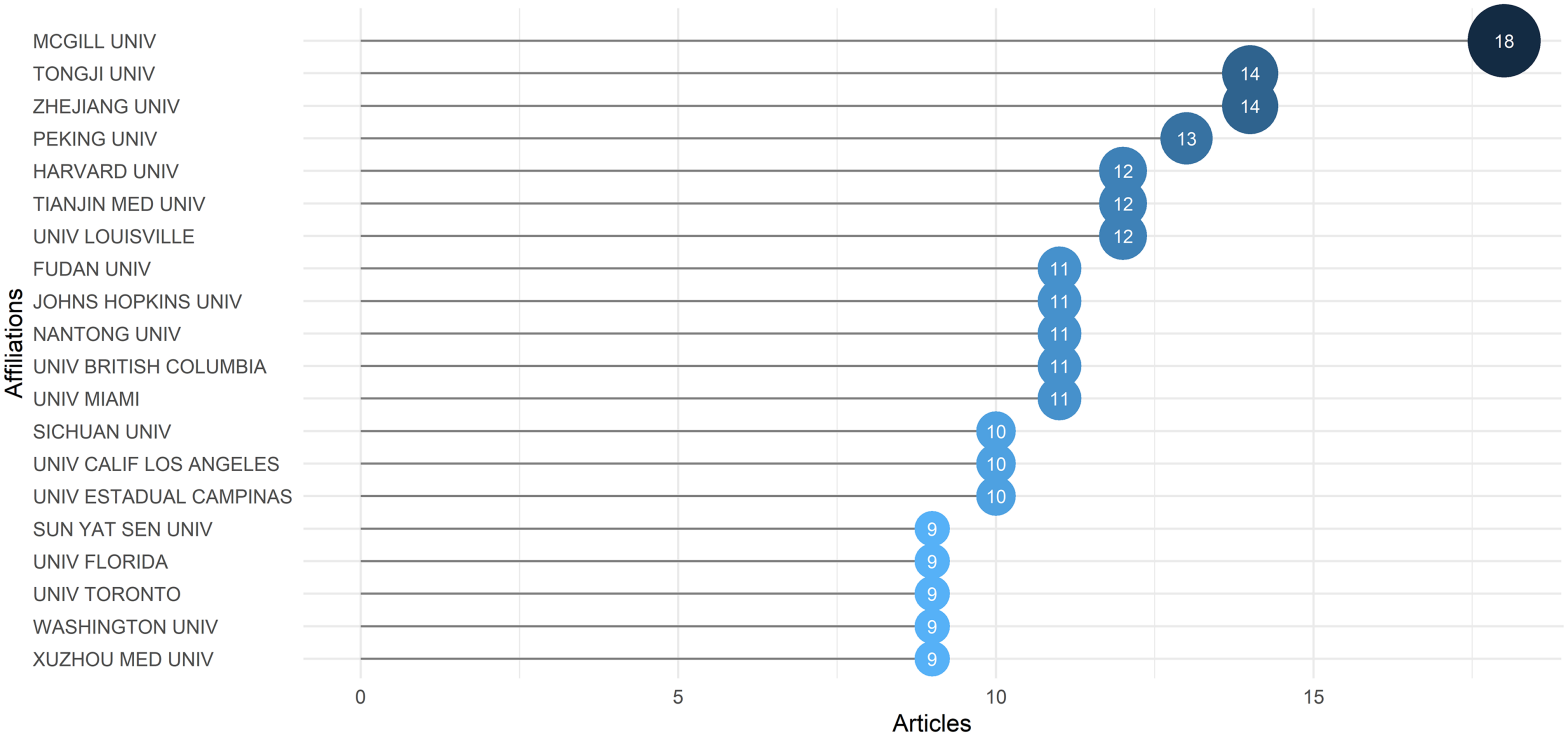
The top 20 affiliations with the most publication output.

Moreover, an examination of author affiliations may reveal cooperation and relationships between academic institutions. The top 20 relevant affiliations in terms of publication output were listed in Figure 4. McGill University took the first place with 18 articles, followed by Tongji University and Zhejiang University with 14 articles each, Peking University (13), Harvard University, Tianjin Medical University and University of Louisville (12). According to the analysis, 9 of 20 productive institutions were from China and 7 from the United States, indicating the exceptional contribution of Chinese and American researchers in the field. We used Citespace to visualize the collaboration network among authors’ affiliations. Supplementary Figure S2C showed that Harvard University, King’s College London, Shanghai Jiao Tong University, and Fujian Medical University were productive and influential, which formed a number of links with other affiliations.

### Journal analysis

**Figure 5:**
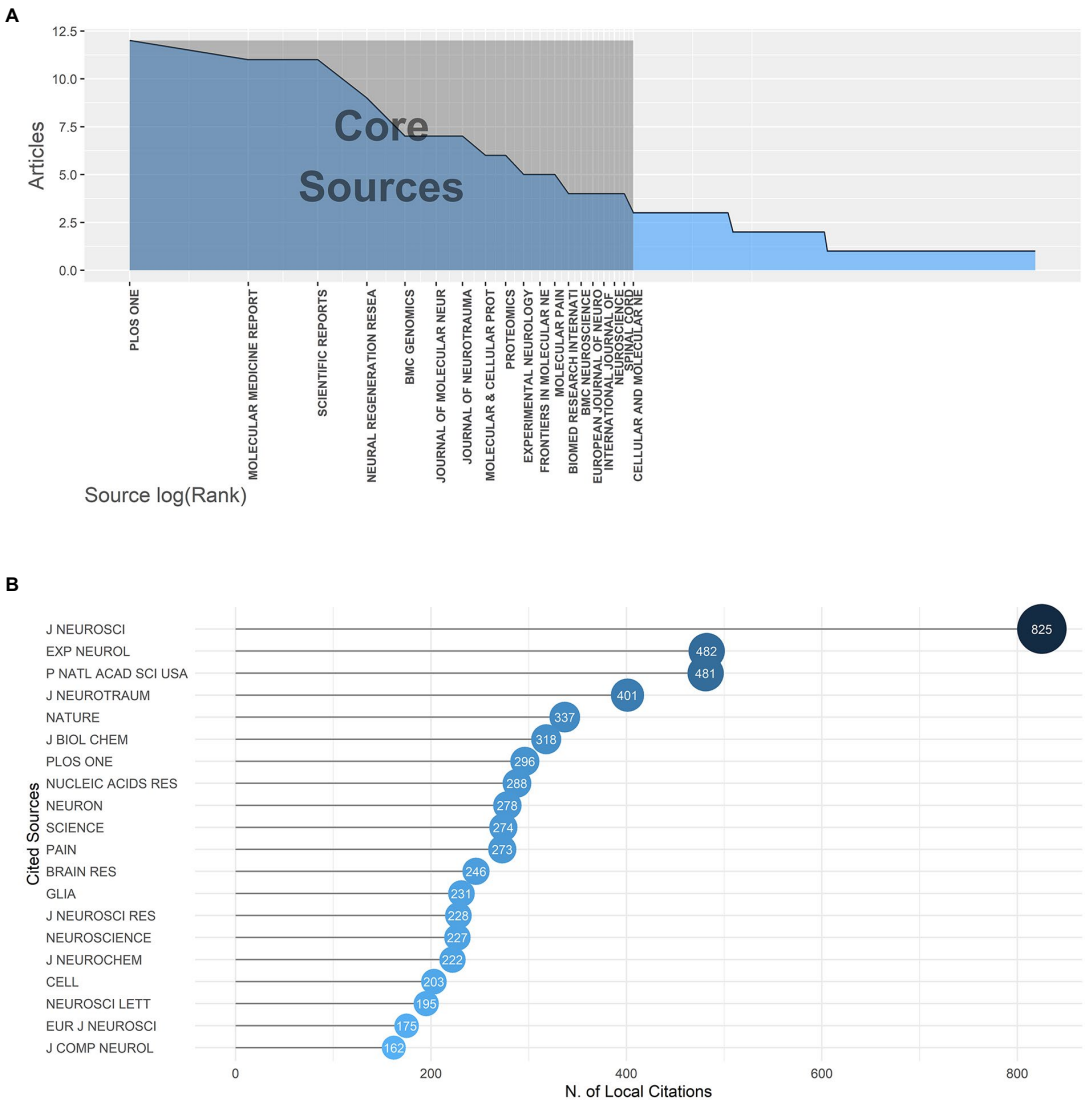
**(A)** Bradford’s law; journals’ orders of arrangement corresponded to their publication outputs. **(B)** The top 20 most locally cited journals.

12 journals with more than five publications on gene expression in SCI from 2000 to 2022 were listed in Supplementary Table S2. These top 12 most popular journals have published 91 articles in total, accounting for 25.93% of all articles included in our study. *Plos One* (IF = 3.240) ranked first with 12 publications and had a dominantly high h-index of 11. Others published 5 to 11 documents with an h-index of 4 to 6. The rankings of publication numbers and h-index were displayed in Supplementary Figures S3A,B. According to Bradford’s law showed in Figure 5A, 19 journals were included in the core journals, and their order of arrangement corresponded to their publications number, which indicates that the core journals in a research field often produced more articles than marginal or relevant journals. In Supplementary Figure S3C, the change of cumulative publications of the top nine journals from 2000 to 2022 showed that *Plos One* publication volume had increased rapidly since 2009 and reached its peak in 2017, which reflected the swift development of emerging journals and huge potential for research on gene expression in SCI.

Figure 5B displays the top 20 most locally cited sources. The publications in the *Journal of Neuroscience* (IF = 6.167) were cited by the retrieved documents 825 times, far more than other journals, which indicated that the *Journal of Neuroscience* was the most influential source in the field. When the top 12 highly productive journals were compared to the top 20 most cited sources, *Experimental Neurology* (IF = 5.330), *Journal of Neurotrauma* (IF = 5.269) and *Plos One* were relatively authoritative and popular in the field of gene expression in SCI. Using Citespace, the most cited journals in this field were also identified: Nature (IF = 69.504), Proceedings of the National Academy of Sciences of the United States of America (IF = 12.779), Journal of Neuroscience (IF = 6.167), Journal of Neurotrauma (IF = 5.269), Experimental Neurology (IF = 5.330). This pattern indicated that the research results in gene expression in SCI were of great significance (Supplementary Figure S3D).

### Documents and references analysis

**Figure 6:**
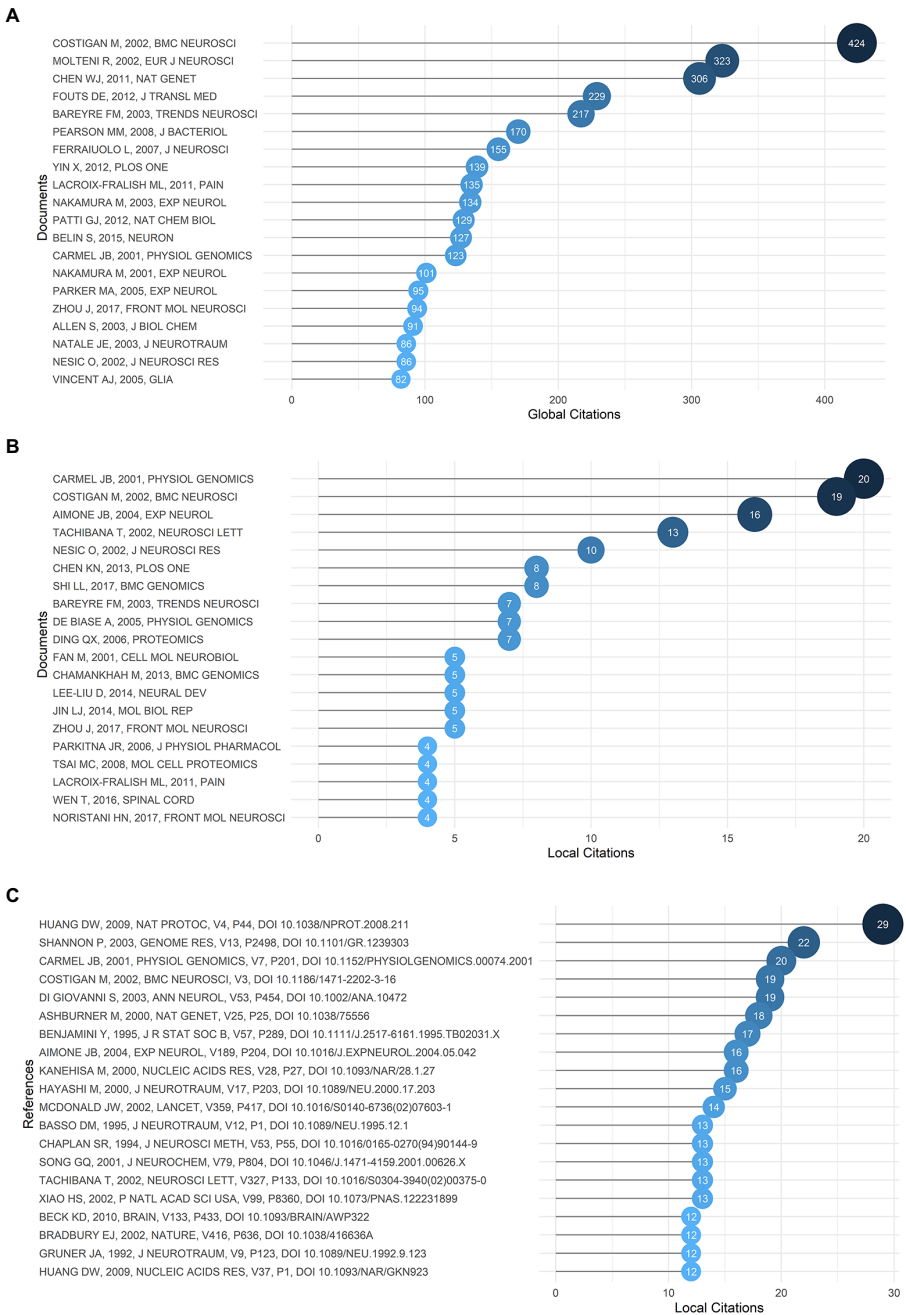
**(A)** The top 20 most globally cited documents. **(B)** The top 20 most locally cited documents. **(C)** The top 20 most locally cited references.

Generally, global citations represent the influence of an article in the whole database, and local citations indicate the impact of a certain article in our retrieval collection. The top 20 global and local cited documents were shown in Figures 6A,B. It was found that *Gene expression profiling of acute spinal cord injury reveals spreading inflammatory signals and neuron loss* (Carmel et al., 2001) and *Replicate high-density rat genome oligonucleotide microarrays reveal hundreds of regulated genes in the dorsal root ganglion after peripheral nerve injury* (Costigan et al., 2002) were the top two most cited documents locally with 20 and 19 citations, and were cited globally 123 and 424 times, respectively, and the latter took first place among the globally cited documents. In addition, both of them were also front-ranking in the most locally cited references shown in Figure 6C, indicating that these two articles might be the cornerstones of the field with enormous significance and were directly cited by other high-impact articles later. The top cited article by Carmel et al. (2001) carried out the first large-scale microarray analysis of gene expression in acute SCI, which inaugurated a novel direction in the field with pioneering implications. The other article by Michael Costigan in 2002 illuminated microarray parameters, contributing to a reduction in errors, which provided strong evidence for subsequent studies. Network analysis of documents by Citespace was displayed in Supplementary Figure S4A, and the top five most cited documents were identified. Amanda Phuong Tran, the author of one of the documents, published an article in 2018 focusing on the biological process of axonal regeneration after SCI in order to reveal target strategies that aided functional recovery and neuroplasticity (Tran et al., 2018). Another study by Shane A. Liddelow suggested that reactive astrocytes were induced after central nervous system injury, which promoted the death of neurons (Liddelow et al., 2017). These highly cited documents were of remarkable significance in that particular field.

Supplementary Figure S4B displayed the documents’ network, in which 250 documents were grouped into six clusters. Documents with similar keywords and subjects would be grouped into the same cluster. And the node size represented the number of references cited by a certain document. Additionally, documents with a high normalized local citation score were considered key documents. One of the highly cited documents in the green cluster illustrated DEGs and key pathways after SCI, which had never been reported before, such as the subunit of GM-CSF receptor, colony stimulating factor 2 receptor beta (Csf2rb), was up-regulated in the acute phase of SCI, which might promote the ligand-receptor binding to strengthen the axonal completeness (Shi et al., 2017). In the red cluster, the key documents reported the changes in expression of circular RNAs (circRNAs) after SCI, detected by microarray and quantitative reverse transcription-PCR (qRT-PCR), and the pathways they affected, such as the AMP-activated protein kinase signaling pathway (Qin et al., 2019). In the cluster colored in yellow, a key document showed the non-coding RNAs (ncRNAs) expression patterns of SCI by sequencing technology and might be involved in the PI3K-Akt signaling pathway related to neuropathic pain (Zhou et al., 2017). In the purple cluster, the fundamental document carried out a bioinformatic analysis on differentially expressed genes (DEGs) in SCI and found the transcription factor ATF3 was up-regulated after SCI and maintained a high level of expression until 28 days after SCI (Guo et al., 2019). Documents in the brown cluster mainly focused on olfactory ensheathing cells (OECs) with the capacity to enhance regeneration of the spinal cord (Guérout et al., 2010; Liu et al., 2010). In the final cluster, it was found that matrix metalloproteinase-8 (MMP-8) protein increased in cerebrospinal fluid (CSF) after SCI with the application of proteomics analysis, and it could become a biomarker of SCI (Light et al., 2012).

### Research hotspots: Keywords network analysis

It is well known that keywords are highly refined and can summarize a certain topic for an article, giving readers a comprehensive overview of the literature. Thus, high-frequency keywords extracted from existing publications by bibliometric methods are used to demonstrate some major issues in a discipline and to find research hotspots in the given field. 1,422 keywords have been extracted from 351 documents and the top 20 most frequent keywords are listed in Supplementary Table S3. The top ranked keyword was “spinal cord injury” with 55 occurrences, followed by “expression” with 46 occurrences and “spinal cord” with 44 occurrences. “Gene expression,” “functional recovery” took fourth and fifth place with 35 and 31 occurrences, respectively. “Brain,” “activation,” “injury,” “messenger RNA,” and “central nervous system” ranked sixth to tenth. CiteSpace was employed to analyze the usage of keywords and to construct a keyword co-occurrence visualization network (Supplementary Figure S5). There were 499 nodes in the keyword network, indicating 499 keywords were utilized in the literature in this field. Additionally, the number of simultaneous appearances of any two keywords in a single document was 2,489. The top 20 keywords in terms of frequency were found in this network. Besides our search terms, the highest ranked keywords, including “neuropathic pain,” “functional recovery,” “central nervous system,” “brain,” “activation,” “protein,” “messenger RNA,” “differentially expressed gene,” “apoptosis,” “inflammation,” and “axonal regeneration” showed the forefront of research on this topic, which may be the future trend of gene expression in SCI. Keywords above extracted by Citespace were similar to those we identified using Biblioshiny, proving the feasibility, accuracy, and effectiveness of our methods, which could also predict the tendency of this field.

**Figure 7:**
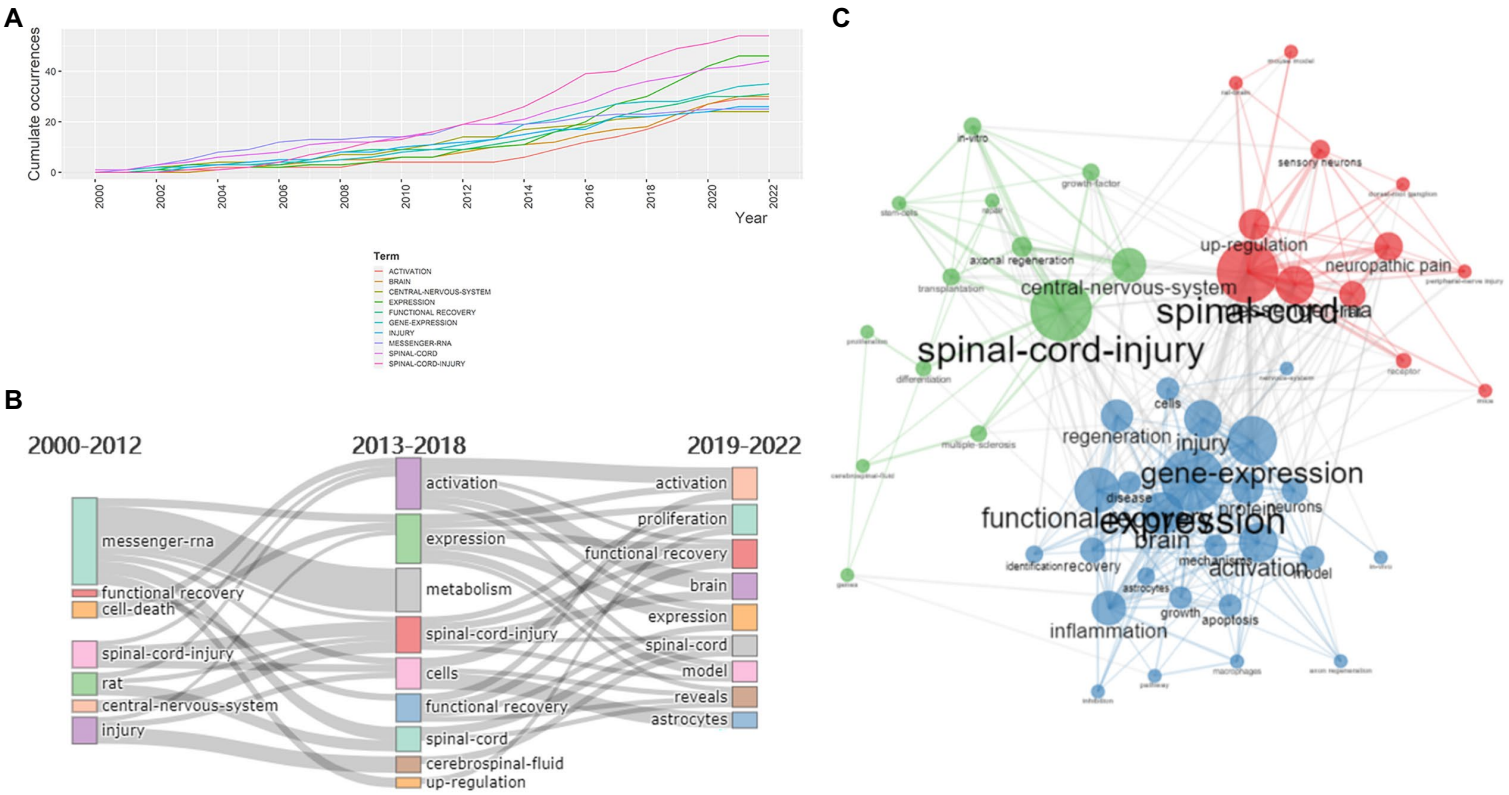
**(A)** The keyword occurrence dynamic from 2000 to 2022. **(B)** The evolution of keywords over three time periods based on their frequency of occurrences. **(C)** Keyword co-occurrence map of 50 words: The three clusters were in red, blue, and green, respectively.

The word dynamics illustrated in Figure 7A reflected the evolution of research hotspots in this field from 2000 to 2022. As one of the subject words, the occurrences of ‘spinal cord injury’ kept growing and reached 54 times in 2022, while ‘expression’ has experienced explosive growth since 2014. In order to detect the keywords receiving varying degrees of attention at each period and which might relate to certain hotspots at different stages, they were displayed based on their frequency of occurrences at each period in Figure 7B. Before 2013, the keywords included ‘messenger RNA’, ‘rat’ and ‘spinal cord injury’, indicating that the study focused on the molecular changes within animal experiments in SCI. From 2013 to 2018, researchers paid more attention to gene expression profiling after SCI, including keywords such as “activation,” “expression,” “metabolism,” “functional recovery,” and “upregulation.” Since 2019, “proliferation,” “activation,” “functional recovery,” and “expression” have received continuous interest, suggesting the downstream mechanism of SCI was another hot topic in the field.

Keywords co-occurrence analysis was applied to explain the inner relationships and degree of betweenness and closeness among keywords, which could help researchers make sense of the frontiers and evolution of the field. Keywords appearing in the same article were related in a network, which formed the keyword co-occurrence map. Closely related keywords were grouped into distinct clusters, which indicated the core research hotspots that the keywords referred to. After excluding the keywords without actual meanings, 50 keywords were selected for drawing the keyword co-occurrence map to visualize the connection and frequency of each keyword (Figure 7C). The nodes with different colors represented the corresponding keywords of different clusters in the map, and the node’s size represented the number of publications with corresponding keywords in this field. As shown in the map, there were three clusters, indicating three research themes or topics, respectively. The red cluster consisted of spinal cord, messenger RNA, neuropathic pain, up regulation, rat, sensory neurons, receptor, mice, peripheral-nerve injury, dorsal-root ganglion, mouse model, and rat brain. The blue cluster included expression, gene expression, functional recovery, brain, activation, injury, regeneration, inflammation, protein, disease, cells, neurons, apoptosis, identification, mechanisms, growth, astrocytes, *in vivo*, macrophages, pathway, and inhibition. The green cluster was composed of spinal cord injury, central nervous system, axonal regeneration, multiple-sclerosis, differentiation, *in vitro*, cerebrospinal fluid, growth factor, stem cells, transplantation, proliferation, and repair. The connected lines between two nodes represented the correlation between them, while some keywords, for instance, spinal cord, expression, activation, up regulation and inflammation, had various linkages with others, suggesting a high degree of betweenness and strong connection with other keywords in the field.

### Trend topics and themes analysis

**Figure 8:**
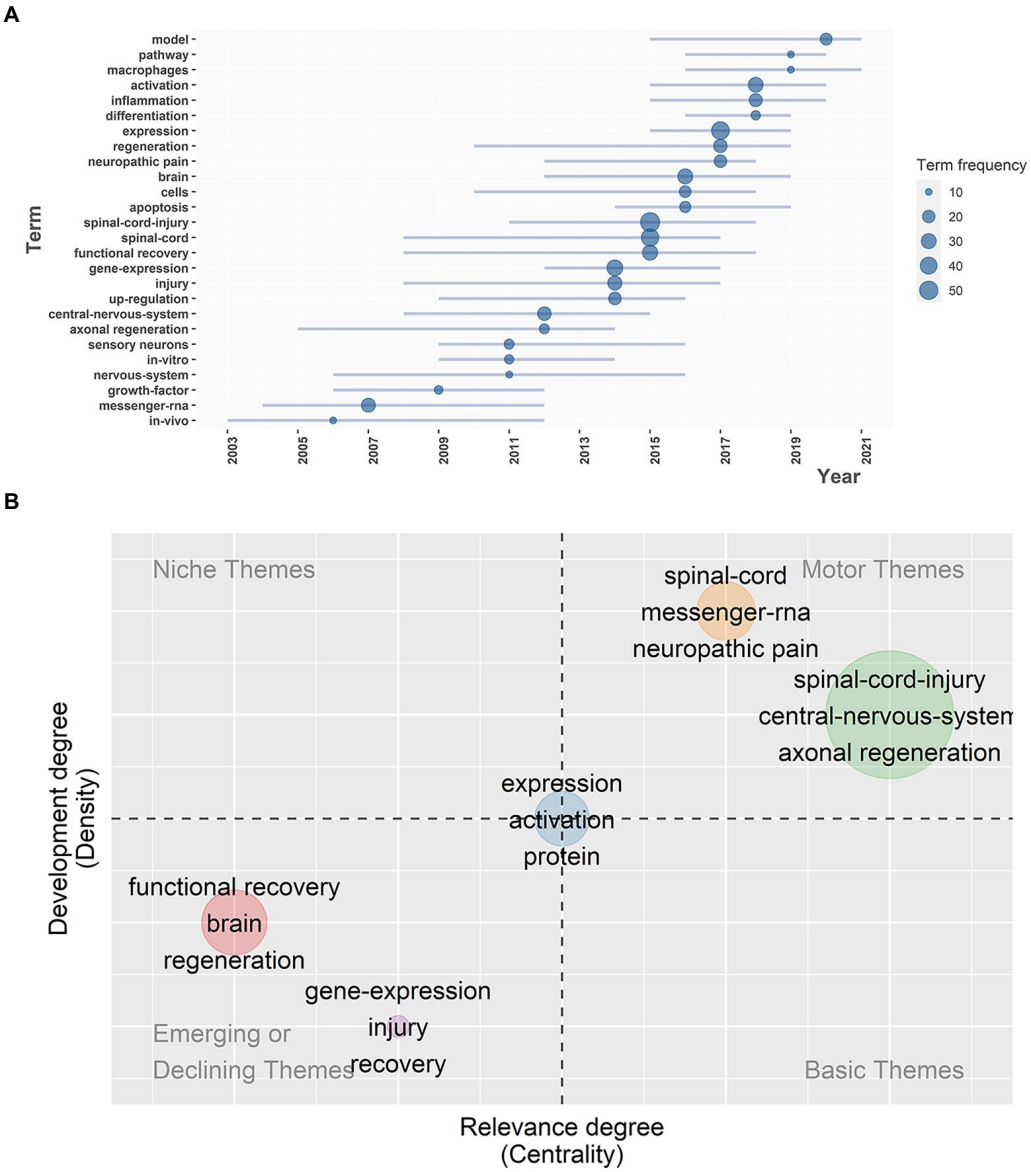
**(A)** The evolution of the most frequently used keywords over time. **(B)** Thematic map; the X axis was centrality while the Y axis was density; the plane was divided into four quadrants, which were motor themes, niche themes, emerging or declining themes, and basic and transversal themes.

To a great extent, trend topics and themes indicate the research potential hotspots and evolution of the field in recent years. According to Figure 8A, terms with a frequency of more than 10 were incorporated into the analysis. From 2011 to 2018, “spinal cord injury” appeared 55 times, while the frequency of “functional recovery” and “gene expression” gradually increased during about the same period, indicating that the gene expression characteristics of SCI have been getting more and more interest during the past decade. In addition, “inflammation,” “activation,” “expression,” “differentiation,” “macrophages,” “pathway.” and “model” emerged around 2015, and have continuously appeared in documents in recent years, especially from 2019 to 2020, which suggests that the seven terms above may become the promising new topics to explore and study in the near future. It was probably due to the introduction of the concept of precision medicine that scholars paid more attention to the molecular and pathological mechanisms of SCI, which provided targets for clinical treatments.

For the purpose of analyzing the development of each theme, a thematic map was drawn in Figure 8B. Its X-axis was centrality, representing the relevance and importance degree of the theme in the field, while its Y-axis was density, representing the development degree. Divided into four quadrants, quadrant 1 (upper right) represented motor themes, which were both important and well developed, and quadrant 2 (upper left) represented niche themes or highly developed and isolated themes with good development but less importance for the current field. Quadrant 3 (bottom left) represented emerging or declining themes, meaning not well developed, perhaps about to disappear or just emerging. Quadrant 4 (bottom right) represented basic and transversal themes, which were important to the field but less developed, referring to elementary concepts. Five theme clusters were distributed in the four quadrants, and the nodes were labeled with keywords. The more frequently the keywords occurred, the bigger the nodes’ sizes became.

Located in quadrant 1, with both high levels of relevance and development, themes about the spinal cord, messenger RNA, neuropathic pain and spinal cord injury, the central nervous system, and axonal regeneration were core as well as essential and have already advanced within the field. It was discovered that the up-regulation of glial fibrillary acidic protein (GFAP) was induced by SCI activated astrocytes and might be an impetus for the persistent onset of neuropathic pain (Kim et al., 2009). Although the relationship between astrocytes and neuropathic pain remained unclear, it was reported that pulsed radiofrequency ablation could alleviate neuropathic pain by reducing the expression of GFAP, which might become a feasible treatment (Zhu et al., 2021). Another study described how inhibition of the PTEN pathway could increase PI3-kinase expression, facilitating axonal regeneration in the central nervous system (Nieuwenhuis and Eva, 2022). In contrast, themes in quadrant 3, like functional recovery, brain and regeneration were less important and developed compared to other themes, or they were just on the upswing and might become new hotspots in the near future. An article showed that bone marrow mesenchymal stem cells (BMSCs) played an important role in neural regeneration after SCI (Lin et al., 2018) by eliciting Schwann cells and 2′, 3′ cyclic nucleotide 3-phosphodiesterase (CNP) positive cells into the injury region, which acted as a structural support for remyelination and neural regeneration after SCI (Ding et al., 2014). Another study reported that electrical neurostimulation was a prospective therapeutic option for SCI (Hofer and Schwab, 2019). Along with the development of basic research and clinical trials, the clinical indications of electrical neurostimulation have become increasingly popular, especially in neuropathic pain and enhancing function recovery after SCI. Themes of expression, activation and protein were of medium importance and development, indicating that these themes would become more and more significant and have great potential with the deepening of research. A proteomic analysis showed highly expressed 11-zinc finger protein and glypican led to the suppression of axon regeneration after SCI (Ding et al., 2006). Glypican was proven to interact with Slit2 in reactive astrocytes to prevent regenerating axons from growing into the injury site (Hagino et al., 2003). The 11-zinc finger protein participated in the inhibition of cell proliferation in cancer cells by regulating the cell cycle, and it was first discovered as an over-expressed gene after SCI, suggesting a probable endogenous mechanism of blocking neural regeneration (Rasko et al., 2001). Recent research has demonstrated that erythropoietin (EPO) can activate the AMP-activated protein kinase (AMPK) pathway to increase autophagy in SCI, which is beneficial to neuroprotection (Wang et al., 2018). Currently, increasing number of scholars in different fields, especially in functional neurosurgery, chronic pain, and rehabilitation, are paying more attention to this field, and related advancements in the field have become greater. Studies on SCI gene expression, biological mechanisms, subsequent regulatory networks, and emerging therapies have sprung up and set the stage for further research. Because of the lack of efficient treatment for SCI patients, it is urgent to figure out the precise molecular and cellular biological variations of SCI in order to search for potential therapeutic targets to prolong patients’ survival time and improve their prognosis as well as quality of life. Analysis of the hot topics and themes changing from 2000 to 2022 can help scholars comprehensively understand the developing process of the field and provide a reference for them to select research topics and to dig into them.

## Discussion

### General information

A total of 351 publications relating to gene expression in SCI research from 2000 to 2022 were retrieved from the WOS, and a bibliometric analysis was carried out based on these documents to help scholars understand the research trends in the current field. Generally, the number of publications annually reflects the popularity of research in a certain field over time. The first large-scale research on gene expression of acute SCI in rats using oligonucleotide microarrays containing 1,200 gene-specific probes was completed to measure the mRNA changes and the temporal and spatial relationships between these functional genes (Carmel et al., 2001). It was found that the level of mRNAs associated with regeneration, such as phosphodiesterase 4 and glia-derived neurite promoting factor increased in the SCI rat model. Since then, gene expression profiling following SCI has come into sight and the number of publications in this field has increased slowly. In 2008, another study investigated the global protein profile of SCI by a proteomics approach and proved that acidic fibroblast growth factor (aFGF) could induce functional recovery as well as down-regulate the proteins that caused inhibition of regeneration of SCI (Tsai et al., 2008), contributing to the further development of the field. With the progress of NGS, RNA-sequencing technology was implemented to detect gene expression during the SCI process, whose data showed high sensitivity and could identify more differential gene expression and was helpful for designing drugs (Chen et al., 2013). From 2010 to 2021, the publication numbers grew year by year and reached 34 in 2021, indicating a promising prospect for gene expression of SCI.

When it comes to authors, Shi-Qing Feng was the most prolific author and had the highest impact by h-index in the field of gene expression in SCI. As the vice president of Tianjin Medical University General Hospital and the president of the 10th International Association of Neurorestoratology (IANR), Shi-Qing Feng focused on SCI and lumbosacral radiculopathy in basic and clinical studies. In 2018, his team published a meaningful study on *Gene* which examined the long noncoding RNAs (lncRNA) and messenger RNAs (mRNA) expression in the immediate phase of SCI in rats by microarray analysis and detected several differential expressions from untreated controls (Zhou et al., 2018). The results might lead to more experimental research and create potential lncRNA and mRNA targets for therapy for SCI in the instantaneous phase. A year later, his team identified the protein profile of Schwann cell-derived exosomes (SCDEs) by proteomic analysis and unveiled the possible proteins involved in SCDEs, which play fundamental roles in neural restoration and pathways, providing strong evidence that SCDEs could become a new therapeutic strategy for SCI (Wei et al., 2019). In his latest research in 2021, the cytokine expression profile in multiple periods after acute SCI in rats was clarified by cytokine array analysis (Li et al., 2021). Erythropoietin (EPO), Fms related tyrosine kinase 3 ligand (Flt-3 l) and CD48 were down-regulated while monocyte chemotactic protein-1 (MCP-1) was up-regulated till day 7 after SCI, which might guide subsequent studies and innovative treatment methods for SCI. These articles published in recent years indicated that the studies on gene expression profiling in SCI were absolutely becoming more and more popular, with great potential and clinical significance, and Shi-Qing Feng and his team have made some achievements in the current field.

The top five most productive countries were China, the United States, Canada, Japan and Germany. China has published 389 documents, followed by the United States with 333 documents. However, in terms of average article citations, China was only cited 13.67 times, much lower than that of the United States (39.65) and other countries like the United Kingdom (37.18), Australia (31.50) and Canada (24.93), suggesting that China was relatively competent but less influential in the field of gene expression of SCI. According to the MCP ratio, China got 0.0569, which was relatively low for high publication outputs, urging China to strengthen international collaboration.

Analysis of the most relevant affiliations helped to understand the cooperative institutes and establish potential cooperation. The most prolific institute was McGill University from Canada, followed by nine institutes from China and 7 from the United States, showing that the research teams of China, the United States and Canada have already made great contributions in the field and more communication and cooperation need to be formed in the near future.

From the perspective of the most relevant and locally cited journals, the *Journal of Neuroscience* was the most influential journal in this field with 825 citations. *Experimental Neurology*, *Journal of Neurotrauma* and *Plos One* also ranked at the front in both the top 12 most productive journals and the top 20 most cited journals, which suggested that these journals served as references for scholars to consult credible literature in this field. Moreover, researchers focused on the molecular mechanism, pathogenesis and gene expression profile in SCI, especially associated with neural regeneration and functional recovery after SCI, which could provide novel ideas for clinical treatment strategy. The research results were published in these impactful journals, attracting academic attention all over the world.

According to high-impact publications, there were five documents cited locally more than 10 times and five documents cited globally more than 200 times, indicating that the field of gene expression in SCI has already made some progress but still needs further development and more research. Among the highly cited documents, *Replicate high-density rat genome oligonucleotide microarrays reveal hundreds of regulated genes in the dorsal root ganglion after peripheral nerve injury* (Costigan et al., 2002) ranked first in the most globally cited documents list with a total of 424 citations, which not only elucidated parameters for microarray analysis to reduce error but also detected a large number of regulated genes with different functions after peripheral nerve injury, providing direction for further research and laying the foundation for the application of microarray analysis in this field. For instance, a newly found up-regulated gene like lysozyme was related to immune response and inflammation after SCI. Another up-regulated gene, CLP36, encoded cytoplasmic protein, which was involved in cell morphogenesis and migration, contributing to axonal regeneration. Of the top 20 most locally cited documents, 10 articles applied microarray analysis methods, which could identify the differential gene expression and up-or down-regulated genes spatially or temporally following SCI. In these studies, it was reported that heat shock 27-kDa protein, epidermal fatty acid-binding protein, calcitonin gene related peptide (CGRP), etc. showed enormous increases while phospholipase C delta 4, GABA transporter 3 and lecithin: cholesterol acyltransferase genes were down-regulated (Tachibana et al., 2002; Aimone et al., 2004; Rodriguez Parkitna et al., 2006; Chamankhah et al., 2013). Since 2013, four articles have used RNA-sequencing methods to identify key pathways and genes associated with regeneration, inflammation and repair, and etc. after SCI, with the purpose of understanding the molecular and pathological mechanisms of SCI. For instance, it was discovered that tribbles pseudokinase 1 (Trib1) was highly expressed in the acute phase of SCI, which might correlate with the differentiation of macrophages in SCI tissues, and through Kyoto Encyclopedia of Genes and Genomes (KEGG) analysis, pathways like ribosome, dopaminergic synapses and oxytocin in SCI were enriched (Shi et al., 2017). According to an RNA-seq analysis of microglia, the molecular mechanisms of microglia shifted from regeneration and neuroprotection in the early stage after SCI to inflammation in a more chronic state (Noristani et al., 2017). Several microglia-derived proinflammatory molecules such as interferon-induced transmembrane protein 1, 2, and 3 increased after injury, which had been reported to restrain neurite growth and affect neurodevelopment (Ibi et al., 2013). Overall, sequencing technology in SCI is becoming increasingly popular. New target genes or transcripts and their contribution to SCI pathophysiology are gradually being revealed, raising the possibility of new treatments for SCI.

### Research hotspots and trend topics

In a particular bibliometric analysis, keyword analysis is one of the most indispensable parts, which reflects the general contents and themes of a certain document and shows the research hotspots, while the keywords’ variation over time represents the evolution of the field. After analyzing the trend topics, word dynamics and thematic map, it was noticed that keywords like spinal cord injury, gene expression and functional recovery continued to gain attention from researchers, indicating that studying the changes in gene expression in SCI to look for potential therapeutic targets for the recovery of sensory and motor functions of the patients was the permanent topic. In recent years, as the occurrences of keywords such as inflammation, activation, pathway, regeneration and neuropathic pain increased, the molecular as well as pathological mechanisms and downstream pathways of SCI became new hot topics that were widely researched. By examining the keyword co-occurrence network more carefully, we identified three clusters, which are displayed in Figure 7C, and we also summarized the development of the hot topics in this field.

The first cluster was colored in red, with keywords like spinal cord, messenger RNA, neuropathic pain, up regulation and rat, which related to the mRNA variation within animal experiments and neuropathic pain after SCI. As one of the common complications of SCI, neuropathic pain is a sensory deficit, leading to long-time loss of daily function and poor prognosis, which affects about 80% of patients with SCI (Hagen and Rekand, 2015; Wang et al., 2018), and it may be accompanied by molecular changes to some degree (Hassler et al., 2014). Existing research suggested that genes connected with activation of the microglia as well as immune responses, such as RT1.Ma, RT1.Mb, complement C1qβ, and tissue inhibitor of metalloproteinase 1 (TIMP-1), performed an up-regulation after SCI, which seemed to be the early markers of neuropathic pain (Rodriguez Parkitna et al., 2006). Additionally, the rising expression of calcitonin gene-related peptide (CGRP) mRNA appeared to maintain the neuropathic pain. It was also demonstrated that SCI caused an alteration in the gene expression of the central nervous system, for instance, the Glycine receptor (GlyR; Esmaeili and Zaker, 2011). A complicated alteration in GlycurrenR mRNA expression level appeared 6 h after SCI, which might play a key role in the progression of neuropathic pain. The subunit GlyRα1 possessed the greatest up-regulation in all regions while GlyRα2 was down-regulated (Esmaeili and Zaker, 2011). GlyRs mainly mediated the neurotransmission negatively in spinal cord and took part in the process of excitotoxic neuronal cell death, which was existed in various neurological disorders (Mayer and Westbrook, 1987). Besides, mRNA expression of preproenkephalin and preprodynorphin increased in response to the injury (Abraham et al., 2000). More research is required to figure out the potential target point for the purpose of relieving neuropathic pain. Currently, studies have shown that salvianolic acid B (SalB) could inhibit the toll-like receptor 4 (TLR4)/myeloid differentiation factor 88 (MyD88) pathway in the SCI model to reduce pain (Wang et al., 2019). This canonical pathway mediated the inflammatory signals, which were closely related to chronic neuropathic pain, and inhibition of the pathway was proven to reduce the pain (Jurga et al., 2016). What’s more, from the thematic map, mRNA and neuropathic pain were motor themes, indicating that they were popular research directions now and in the near future. More insightful studies on neuropathic pain itself and its pathophysiology are expected, in order to uncover hidden molecules and enact subsequent treatment.

The second blue cluster was centered on expression, functional recovery, activation, regeneration, inflammation, apoptosis and pathway, related to the molecular and pathological mechanisms and the downstream pathway of SCI. SCI pathological mechanisms include vascular alteration such as hemorrhage and ischemic necrosis, metabolic disturbances, free radicals accumulation, excessive excitatory neurotransmitter production, cellular inflammatory response and neuron and neuroglia apoptosis (Oyinbo, 2011; Couillard-Despres et al., 2017; O’Shea et al., 2017; Anjum et al., 2020). However, so far, the detailed and accurate pathological mechanisms of SCI have not been clarified yet, and thus it is an important issue to illuminate the underlying mechanism in order to explore more molecule or protein targets for novel efficient therapy for SCI, improving the quality of patients’ lives. A study using DNA microarrays has been carried out to observe the gene expression changes after SCI, with up-regulated genes associated with transcription, inflammation, like IL-6, tumor necrosis factor (TNF), and down-regulated genes involved in neurotransmission in the acute phase, and the increased expression of extracellular matrix molecules, growth factors and angiogenic factors in the later phase (Bareyre and Schwab, 2003). It was confirmed in SCI model rats that nerve growth factor inhibited the apoptosis induced by endoplasmic reticulum stress in the central nervous system, which improved functional recovery at the same time, suggesting a trend of nerve growth factor translational drug research in this field (Zhang et al., 2014; Xu et al., 2019). Another research identified the transcriptome of SCI in acute and subacute stages and discovered several enriched pathways by RNA-seq, for instance, ‘Role of Pattern Recognition Receptors in Recognition of Bacteria and Viruses’ and ‘Atherosclerosis Signaling’ pathways (Chen et al., 2013). The former pathway was associated with inhibition of apoptosis, involving C3ar and C5ar, while the latter pathway, containing Cxcr4 and Ccr2, contributed to neuropathic pain and regulation of neural repair processes, since the suppression of Cxcr4 could improve re-myelination and functional recovery (Carbajal et al., 2011; Van Steenwinckel et al., 2011). Currently, various materials have been identified and developed that target the pathological mechanisms and multiple pathways of SCI. Curcumin treatment has been upheld to promote functional recovery, decrease neuron apoptosis and suppress the inflammatory response after SCI (Li et al., 2021). By inhibiting the Akt/mTOR signaling pathway, it exerted a neuroprotective effect and induced autophagy flux after SCI, which was reported to be beneficial for motor function recovery, decrease of tissue damage, and neuronal death rate (Heras-Sandoval et al., 2014; Nikoletopoulou et al., 2015). Another substance, quercetin, could promote locomotor function recovery and astrocyte activation, contributing to the axonal regeneration through BDNF and JAK2/STAT3 pathways in SCI (Wang et al., 2018). Generally speaking, despite the fact that a variety of studies have yielded some success in this perspective, there are still more research points needed to investigate. On the basis of existing studies on biological mechanisms, future researchers can refer to these existing ideas and illuminate them through advanced sequencing technology to obtain clear and clinically significant conclusions.

Colored in green, the third cluster contained items like spinal cord injury, central nervous system, differentiation, transplantation and repair, which represented the emerging viable treatments for the disease. Due to the lack of effective treatments for SCI, the transplantation of cells with the ability to repair central nervous system injuries into the spinal cords of patients is a popular research direction, which can promote the regeneration and recovery of the spinal cord. In a recent study, transcriptomics and non-quantitative proteomics methods were applied to identify the gene and protein profiles of olfactory ensheathing cells (OECs) from the olfactory mucosa (OM) as well as the olfactory bulb (OB), which both had the ability to repair nerve injury (Lan et al., 2022). OM-derived OECs expressed genes and proteins related to cytokine binding, regulation of inflammatory response and wound healing. OB-derived OECs were closely connected with regulation of axon regeneration and transmission of nerve impulses. For instance, when overexpressed by OM, tumor necrosis factor receptor superfamily member 25 (Tnfrsf25) played a key role in the inflammatory course and regulation of apoptosis processes. It expanded the number of regulatory T cells to enhance the immune response against the inflammation caused by nerve injury (Copsel et al., 2020). Neurotrophic receptor tyrosine kinase 2 (Ntrk2) was highly expressed by OB and could promote axonogenesis and neuron projection development (Pattwell et al., 2020). Therefore, the transplantation of OECs was a feasible therapeutic strategy for SCI treatment, which not only helped restore injured nerve function partially but also facilitated neural regeneration. Besides, based on the characteristics of self-renewal and differentiation, the utilization of neural stem cell (NSC) transplantation to treat SCI is viable, and elucidating the role of interesting molecules within the NSC differentiation process may provide candidate targets to induce appropriate differentiation and promote the application of NSC in the clinic, for example, miR-31 (Wei et al., 2010). After analyzing the expression profile of mRNA and miRNA between the miR-31 overexpression group and the controls by RNA-seq technology, researchers concluded that miR-31 was responsible for maintaining the stemness of NSC and inhibiting the differentiation of NSC into motor neurons, while restraining miR-31 helped to increase the proportion of generated functional neurons, accelerating the process of spinal cord repair (Li et al., 2020). In addition to OECs and NSCs, neural progenitor cells, oligodendrocyte precursor cells, and mesenchymal stem cells can also be used for transplantation (Assinck et al., 2017). Understanding the mechanisms whereby the above cells promote damage repair by sequencing technology will be beneficial to make cell transplantation a noteworthy clinical choice and develop more targeted therapies in the near future, which can absolutely improve the prognosis of individuals with SCI (Tetzlaff et al., 2011). Further studies can be performed in the direction of single-cell sequencing of SCI to explore deeper mechanisms and structures in order to localize key genes in specific cells and illuminate their functions. The integration of single-cell sequencing and bulk multi-omics sequencing of tissues is also recommended to construct regulatory networks of transcription factors, targeted genes, downstream pathways and phenotypes of SCI, which provides evidence for the development of treatments.

### Limitations

This study summarized the publications from 2000 to 2022 as the first bibliometric analysis of documents concerning gene expression in SCI. Nonetheless, flaws cannot be avoided because of the data acquisition method. We only retrieved the WOS Core Collection database so that the relevant publications may be incomplete. We can integrate the documents from multiple databases to obtain data more comprehensively in further research. Additionally, the articles published after the date we retrieved them could not be included in our analysis, resulting in a lack of timeliness. Besides, we should communicate with influential scholars in the field to improve our objective cognition and increase our knowledge of frontiers.

## Conclusion

In this study, we applied bibliometric analysis to summarize knowledge on gene expression in SCI and generated a visualization of collected data. Apart from identifying the most prolific and influential authors, countries, affiliations and core journals in the field, we focused more on three research hotspots and trend topics. The core of each hotspot, such as neuropathic pain, gene expression, regeneration, transplantation, and functional recovery, reflected that the molecular and pathological mechanisms and potential treatments for SCI were popular and promising directions, which might be the future trend of this field. This study helps scholars understand the evolution of the field of gene expression in SCI and provides references for scholars in choosing research topics, especially the cutting-edge developments. The analysis of core journals and authors also guides them to select journals for submission or researchers to cooperate with.

## Data availability statement

The original contributions presented in the study are included in the article/Supplementary material. Further inquiries can be directed to the corresponding authors.

## Author contributions

SW, WQ, SC, SX, MJ, YL, HZ, HQ, XZ, JZ, XY, CS, PY, RH, and ZH: conception/design, collection and/or assembly of data, data analysis and interpretation, manuscript writing, and final approval of manuscript. All authors contributed to the article and approved the submitted version.

## Funding

This study was supported in part by the Henan Medical Science and Technology Research project (no. 201602031); Key Project of Provincial and Ministerial Co-Construction of Henan Medical Science and Technology (no. SBGJ202002031). The funders had no role in study design, data collection and analysis, decision to publish, or preparation of the manuscript.

## Conflict of interest

The authors declare that the research was conducted in the absence of any commercial or financial relationships that could be construed as a potential conflict of interest.

## Publisher’s note

All claims expressed in this article are solely those of the authors and do not necessarily represent those of their affiliated organizations, or those of the publisher, the editors and the reviewers. Any product that may be evaluated in this article, or claim that may be made by its manufacturer, is not guaranteed or endorsed by the publisher.
