# Supplementary material for: Bibliometric analysis of research on gene expression in spinal cord injury
Source: Front Mol Neurosci. 2022 Oct 31;15:1023692. doi: 10.3389/fnmol.2022.1023692 (PMC9661966; doi:10.3389/fnmol.2022.1023692)
Supplement: Supplementary file 1 [file Table_1.DOCX]

Table S1. The top 8 most contributing countries

| Rank | region | Records | Total Citations | Average Article Citations |
| --- | --- | --- | --- | --- |
| 1 | CHINA | 389 | 1682 | 13.67 |
| 2 | USA | 333 | 3529 | 39.65 |
| 3 | CANADA | 75 | 349 | 24.93 |
| 4 | JAPAN | 46 | 291 | 18.19 |
| 5 | GERMANY | 41 | 184 | 16.73 |
| 6 | UK | 40 | 409 | 37.18 |
| 7 | FRANCE | 31 | 144 | 16 |
| 8 | AUSTRALIA | 28 | 189 | 31.5 |
