# Supplementary material for: Bibliometric analysis of research on gene expression in spinal cord injury
Source: Front Mol Neurosci. 2022 Oct 31;15:1023692. doi: 10.3389/fnmol.2022.1023692 (PMC9661966; doi:10.3389/fnmol.2022.1023692)
Supplement: Supplementary file 2 [file Table_2.DOCX]

Table S2. The top 12 highly-productive journals

| Rank | Sources | Number of publications | h-index |
| --- | --- | --- | --- |
| 1 | PLOS ONE | 12 | 11 |
| 2 | MOLECULAR MEDICINE REPORTS | 11 | 4 |
| 3 | SCIENTIFIC REPORTS | 11 | 6 |
| 4 | NEURAL REGENERATION RESEARCH | 9 | 4 |
| 5 | BMC GENOMICS | 7 | 5 |
| 6 | JOURNAL OF MOLECULAR NEUROSCIENCE | 7 | 4 |
| 7 | JOURNAL OF NEUROTRAUMA | 7 | 6 |
| 8 | MOLECULAR & CELLULAR PROTEOMICS | 6 | 6 |
| 9 | PROTEOMICS | 6 | 6 |
| 10 | EXPERIMENTAL NEUROLOGY | 5 | 5 |
| 11 | FRONTIERS IN MOLECULAR NEUROSCIENCE | 5 | 4 |
| 12 | MOLECULAR PAIN | 5 | 4 |
