# Supplementary material for: Bibliometric analysis of research on gene expression in spinal cord injury
Source: Front Mol Neurosci. 2022 Oct 31;15:1023692. doi: 10.3389/fnmol.2022.1023692 (PMC9661966; doi:10.3389/fnmol.2022.1023692)
Supplement: Supplementary file 3 [file Table_3.DOCX]

Table S3. The top 20 most frequent keywords

| Rank | Keywords | Frequency | | | Percentage, % | | Cumulative percentage, % |
| --- | --- | --- | --- | --- | --- | --- | --- |
| 1 | spinal cord injury | | 55 | 3.87 | | 3.87 | |
| 2 | expression | | 46 | 3.23 | | 7.10 | |
| 3 | spinal cord | | 44 | 3.09 | | 10.20 | |
| 4 | gene expression | | 35 | 2.46 | | 12.66 | |
| 5 | functional recovery | | 31 | 2.18 | | 14.84 | |
| 6 | brain | | 30 | 2.11 | | 16.95 | |
| 7 | activation | | 29 | 2.04 | | 18.99 | |
| 8 | injury | | 26 | 1.83 | | 20.82 | |
| 9 | messenger rna | | 25 | 1.76 | | 22.57 | |
| 10 | central nervous system | | 24 | 1.69 | | 24.26 | |
| 11 | regeneration | | 23 | 1.62 | | 25.88 | |
| 12 | inflammation | | 22 | 1.55 | | 27.43 | |
| 13 | protein | | 22 | 1.55 | | 28.97 | |
| 14 | neuropathic pain | | 20 | 1.41 | | 30.38 | |
| 15 | up regulation | | 20 | 1.41 | | 31.79 | |
| 16 | recovery | | 19 | 1.34 | | 33.12 | |
| 17 | disease | | 18 | 1.27 | | 34.39 | |
| 18 | model | | 18 | 1.27 | | 35.65 | |
| 19 | cells | | 17 | 1.20 | | 36.85 | |
| 20 | neurons | | 17 | 1.20 | | 38.05 | |
