# Supplementary material for: Bibliometric analysis of research on gene expression in spinal cord injury
Source: Front Mol Neurosci. 2022 Oct 31;15:1023692. doi: 10.3389/fnmol.2022.1023692 (PMC9661966; doi:10.3389/fnmol.2022.1023692)
Supplement: Supplementary file 4 [file Data_Sheet_1.docx]

**Supplementary Figures**

**
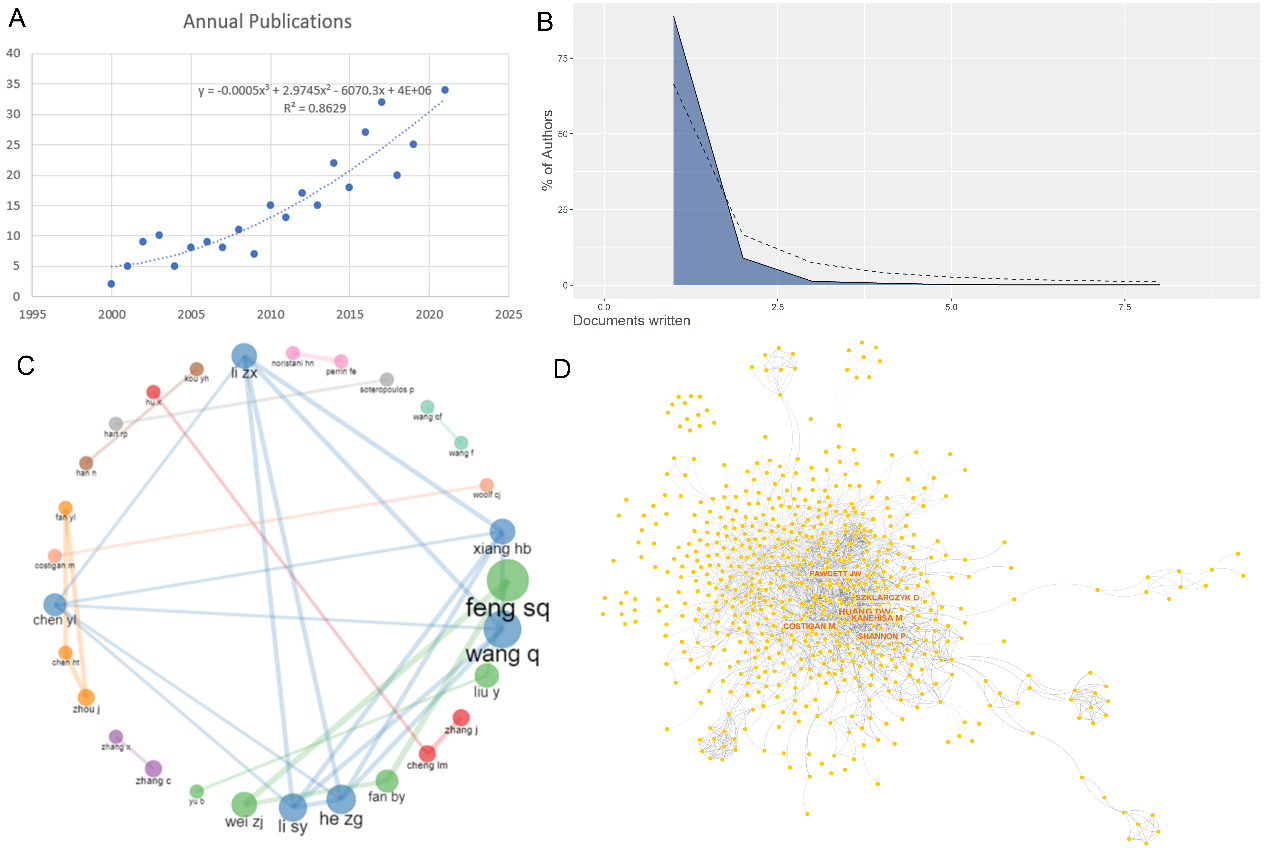
**

**Figure S1. (A)** The estimated annual publication by polynomial model fitting. **(B)** Lotka’s law. The frequency distribution of the scientific productivity of authors. **(C)** The author collaboration network. **(D)** 6 influential authors in this field and the collaboration network created by Citespace.

**
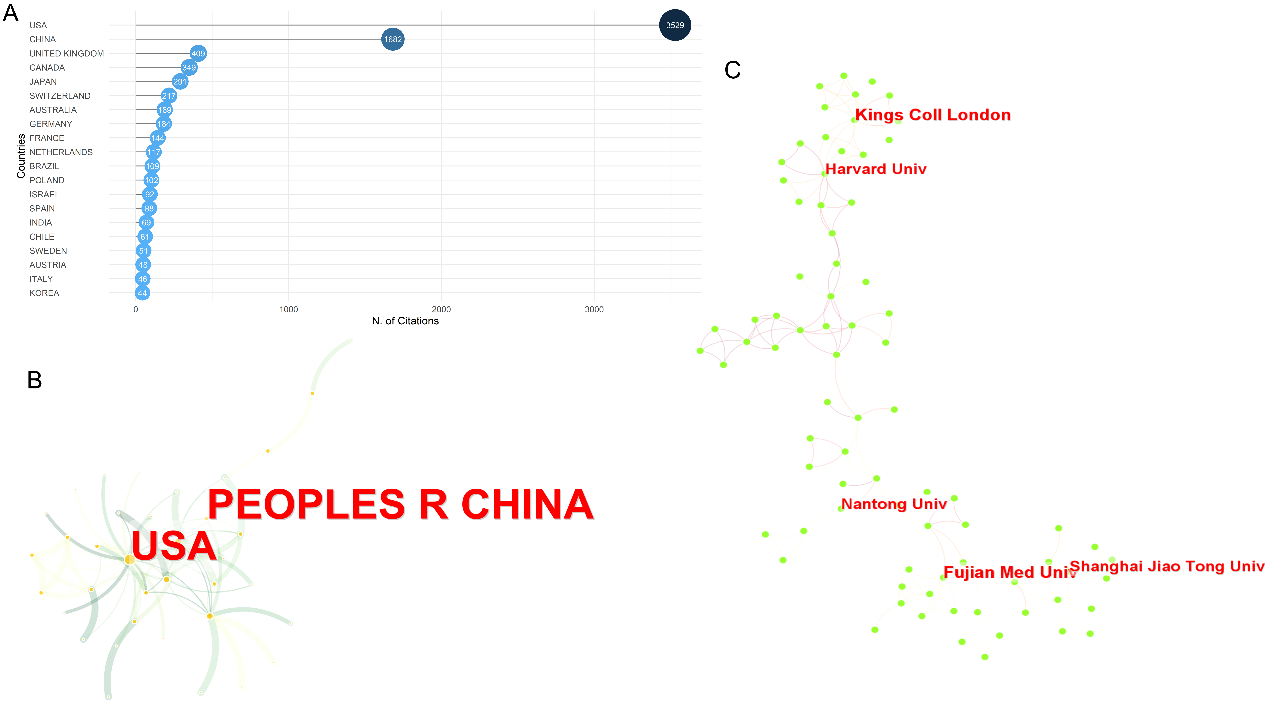
**

**Figure S2. (A)** The top 20 countries in terms of citations. **(B)** The most productive countries and the communications among them created using Citespace. **(C)** The most impactful affiliations and the interaction network of them created by Citespace.

**
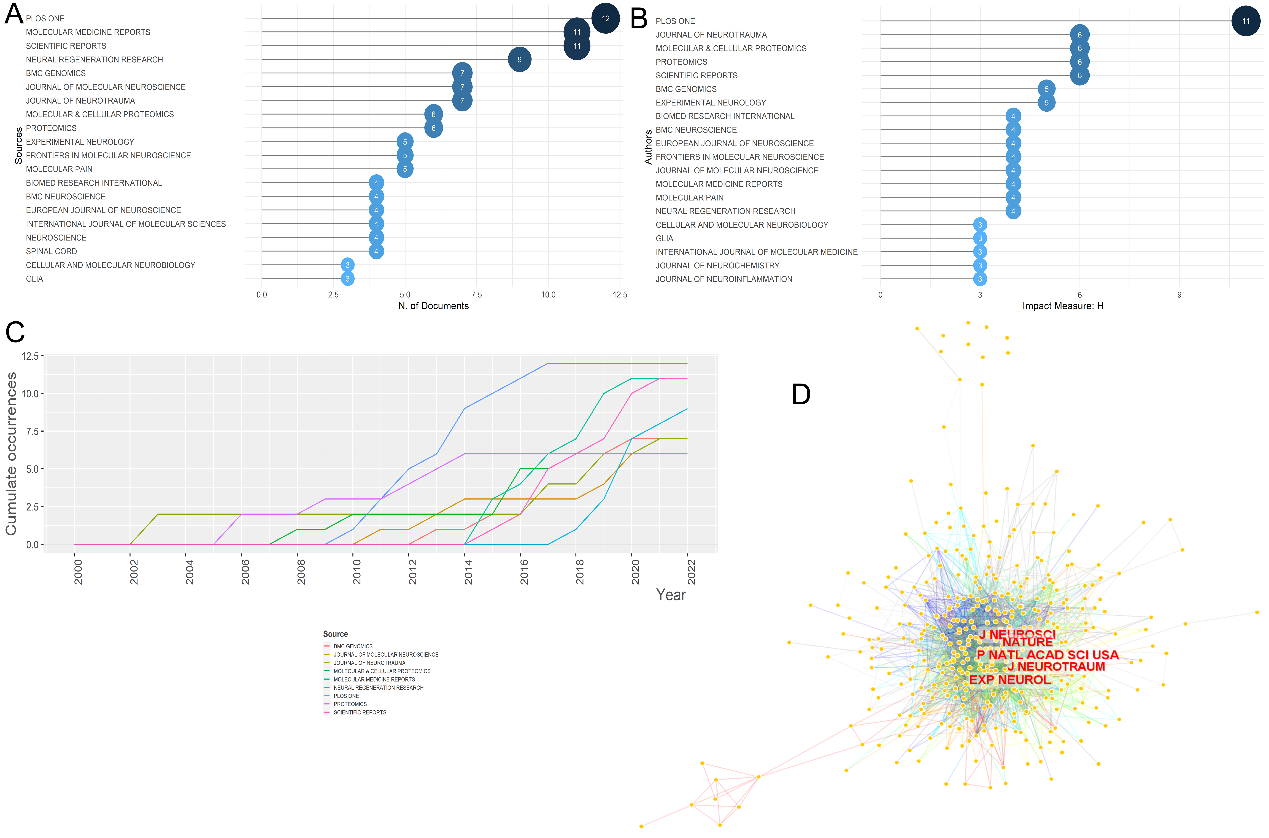
**

**Figure S3. (A)** The top 20 most pertinent sources. **(B)** The sources’ impact is measured by the h-index. **(C)** The dynamic alteration of cumulative publications of the top 9 sources from 2000 to 2022. **(D)** The most cited journals created by Citespace.

**
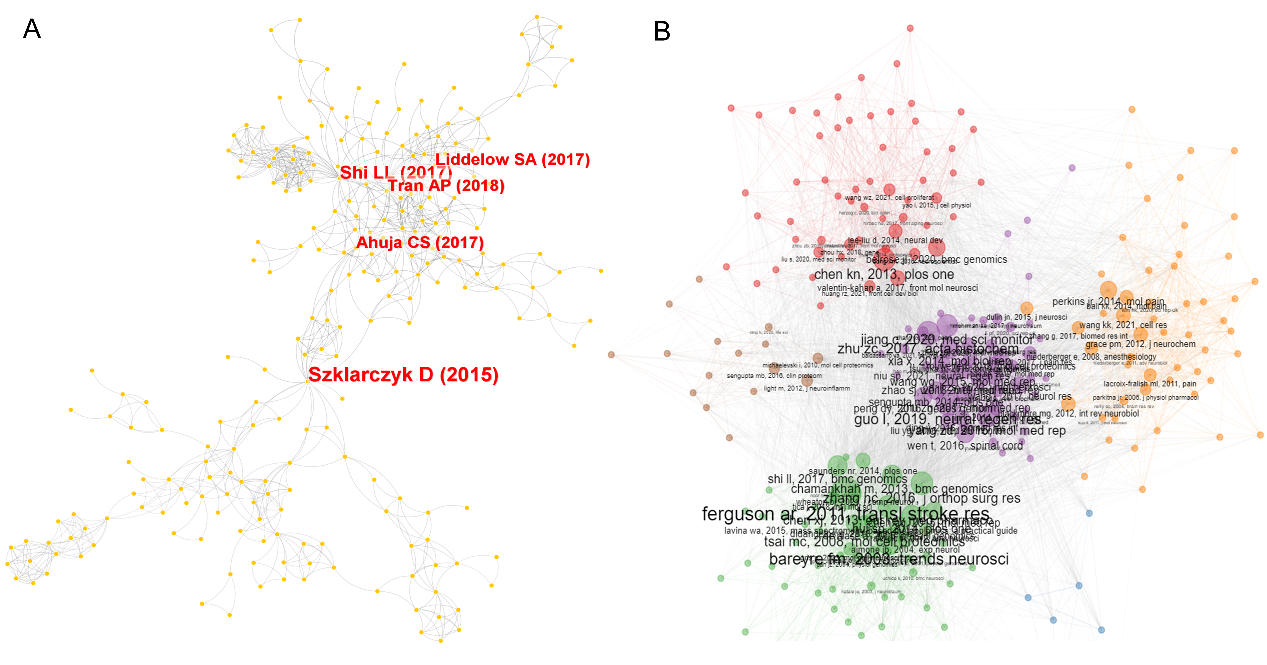
**

**Figure S4.** **(A)** The top 5 most cited documents identified using Citespace. **(B)** The coupling network of 250 documents. Documents with similar keywords and subjects were grouped into the same cluster. Each node represented a document, and the size of the node reflected the number of references cited by this document.


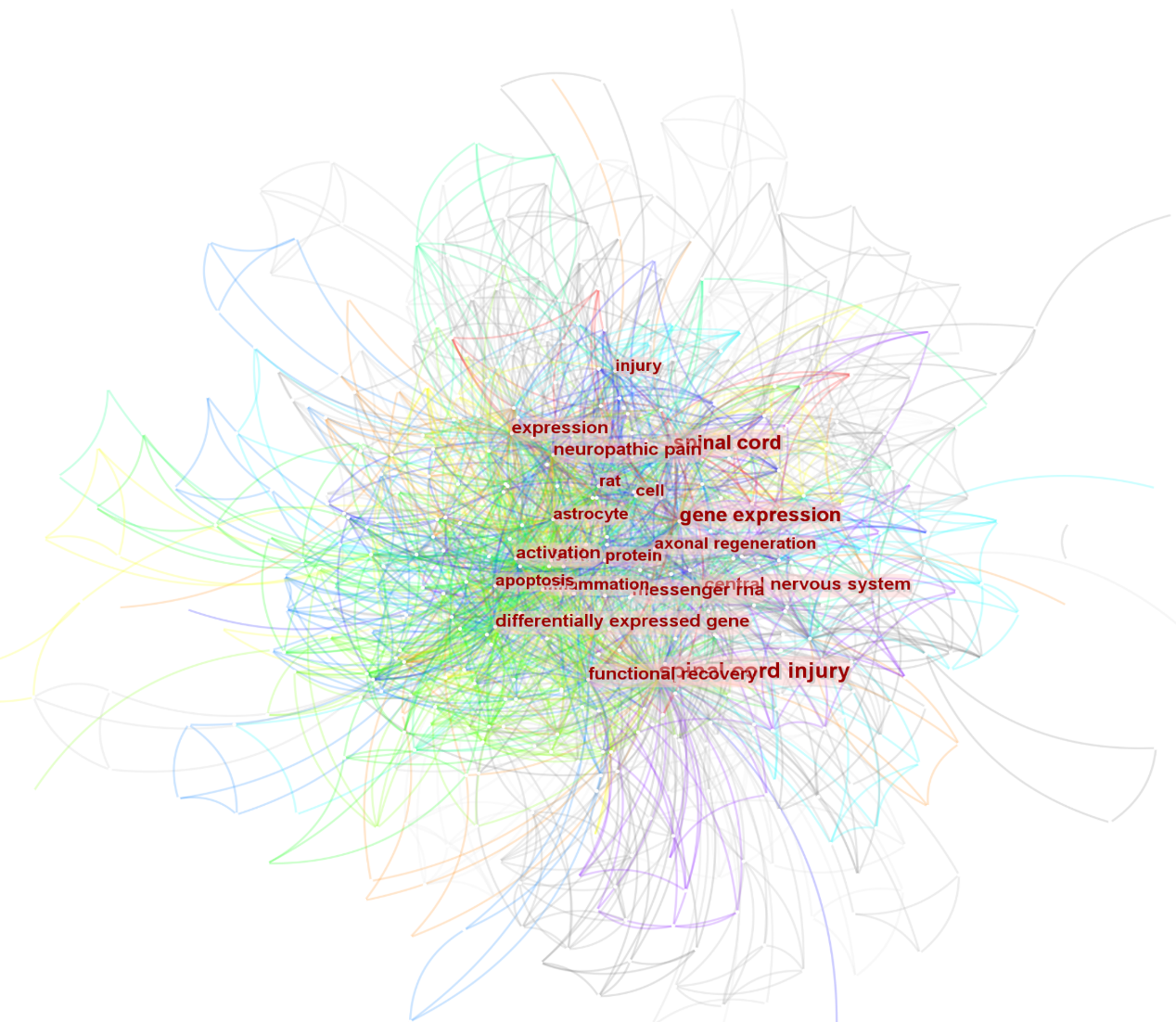


**Figure S5.** The frequency of the usage of keywords and the construction of a keyword co-occurrence visualization network by Citespace.
