# Supplementary material for: Bibliometric analysis of research on gene expression in spinal cord injury
Source: Front Mol Neurosci. 2022 Oct 31;15:1023692. doi: 10.3389/fnmol.2022.1023692 (PMC9661966; doi:10.3389/fnmol.2022.1023692)
Supplement: Supplementary file 6 [file Data_Sheet_3.docx]

The code for our R tools for conducting analysis and visualization is as follows:

install.packages("bibliometrix", dependencies = TRUE)

library(bibliometrix)

biblioshiny()

Steps to reproduce

1. Use R package “bibliometric” with code of “biblioshiny()” to generate an interactive web interface. 2. Click on “Data” and then “Import or Load files”, select “import raw file(s)” with “Database” option of “Web of Science (WoS/Wok)” and File format of “Plain Text”. Upload this data files and then click “Start”. 3. All the functions are available in the interactive web interface.
